# Sleep Quality and Cognitive Decline: A Cross-Country Comparison Between England and China

**DOI:** 10.1093/geroni/igab046.460

**Published:** 2021-12-17

**Authors:** Laura Brocklebank, Dorina Cadar, Li Yan, Yaohui Zhao, Andrew Steptoe

**Affiliations:** 1 University College London, London, England, United Kingdom; 2 Peking University, Guangzhou, Guangdong, China (People's Republic)

## Abstract

Too little or too much sleep is associated with accelerated cognitive decline in older adults. However, sleep duration does not capture other sleep problems prevalent in older adults, such as difficulties with falling or staying asleep. Less is known about the impact of sleep quality on cognitive ageing, and if this relationship differs between England and China. Therefore, the aim of this study is to examine the relationship of self-reported sleep quality with cognitive performance and rate of change over 6-7 years follow-up in two nationally-representative samples of English and Chinese older adults. The primary outcome was a memory score (range 0-20), which was assessed using immediate and delayed 10-word recall tests in both cohorts. The results of bivariate descriptive analyses at baseline suggest there may be an inverted U-shaped association between sleep quality and memory in English older adults, and a positive dose-response association in Chinese older adults.

